# Stromal Cell-Derived Factor-1/CXCL12 Contributes to MMTV-Wnt1 Tumor Growth Involving Gr1^+^CD11b^+^ Cells

**DOI:** 10.1371/journal.pone.0008611

**Published:** 2010-01-19

**Authors:** Bob Y. Liu, Irina Soloviev, Peter Chang, John Lee, XiaoDong Huang, Cuiling Zhong, Napoleone Ferrara, Paul Polakis, Chie Sakanaka

**Affiliations:** 1 Research Oncology, Genentech Inc, South San Francisco, California, United States of America; 2 Physiological Chemistry, Genentech Inc, South San Francisco, California, United States of America; Roswell Park Cancer Institute, United States of America

## Abstract

**Background:**

Histological examinations of MMTV-Wnt1 tumors reveal drastic differences in the tumor vasculature when compared to MMTV-Her2 tumors. However, these differences have not been formally described, nor have any angiogenic factors been implicated to be involved in the Wnt1 tumors.

**Methodology/Principal Findings:**

Here, we show that MMTV-Wnt1 tumors were more vascularized than MMTV-Her2 tumors, and this correlated with significantly higher expression of a CXC chemokine, stromal cell-derived factor-1 (SDF1/CXCL12) but not with VEGFA. Isolation of various cell types from Wnt1 tumors revealed that SDF1 was produced by both tumor myoepithelial cells and stromal cells, whereas Her2 tumors lacked myoepithelial cells and contained significantly less stroma. The growth of Wnt1 tumors, but not Her2 tumors, was inhibited by a neutralizing antibody to SDF1, but not by neutralization of VEGFA. Anti-SDF1 treatment decreased the proportion of infiltrating Gr1^+^ myeloid cells in the Wnt1 tumors, which correlated with a decrease in the percentage of endothelial cells. The involvement of Gr1^+^ cells was evident from the retardation of Wnt1 tumor growth following *in vivo* depletion of these cells with an anti-Gr1-specific antibody. This degree of inhibition on Wnt1 tumor growth was comparable, but not additive, to the effect observed with anti-SDF1, indicative of overlapping mechanisms of inhibition. In contrast, Her2 tumors were not affected by the depletion of Gr1^+^ cells.

**Conclusions/Significance:**

We demonstrated that SDF1 is important for Wnt1, but not for HER2, in inducing murine mammary tumor and the role of SDF1 in tumorigenesis involves Gr1^+^ myeloid cells to facilitate growth and/or angiogenesis.

## Introduction

Mutations in components of canonical Wnt signaling pathway are involved in a variety of human cancers (reviews [1], [2]). This transforming potential is borne out in mouse models where overexpression of Wnt1 or a stable form of the intracellular signaling effector, β-catenin (ΔN89β-catenin or ΔNβcat), under the control of the mouse mammary tumor virus (MMTV) long-terminal repeat induces mammary adenocarcinoma [3]–[5]. Although the mechanism by which Wnt signaling effectors induce mammary tumorigenesis remains unclear, these tumors harbor distinguishing histological features when compared to other mammary tumors induced by overexpression of Her2 or Polyoma middle T antigen (PyMT) driven by the same MMTV promoter. First, Wnt signaling-induced tumors are composed of both ductal luminal cells and contractile myoepithelial cells, whereas Her2 and PyMT tumors contain only luminal cells. Second, a significant portion of the Wnt1 tumors is comprised of stroma, but stroma is minimal in the Her2 tumors [6]. Finally, Wnt1 tumors are frequently filled with blood and lymphocytic infiltrates [3], [6], whereas Her2 tumors are generally pale and devoid of obvious blood-filled regions. These observations suggest that tumor-initiating cells, stromal-epithelial interactions, and tumor vascularization should be obviously different between Wnt1 and Her2 tumors.

Tumor angiogenesis is a rate limiting step in tumor growth, and neo-vascularization can be achieved by a number of ways. Newly forming blood vessels are often sprouted from pre-existing vessels involving the dissociation, migration, and division of differentiated endothelial cells. Micro-vessels can also be synthesized de novo by contributions of bone marrow-derived cells (BMDCs). Various BMDCs such as endothelial progenitor cells, tumor-associated macrophages, Tie2-expressing monocytes, and myeloid progenitor cells, have been shown to participate in tumor angiogenesis and facilitate tumor growth [7]–[13]. Recent studies have shown that Wnt1 tumors recruit BMDCs to the tumor site, and these progenitor cells can be incorporated into the stroma, possibly contributing to tumor angiogenesis [14]. Some BMDCs express high levels of CXCR4 receptor and can be mobilized from the bone marrow to sites producing the chemokine ligand, SDF1 [15], [16]. The importance of the SDF1-CXCR4 axis in angiogenesis is apparent from the lack of gastrointestinal blood vessels in CXCR4^−/−^ mice, and SDF1 has been shown to contribute to angiogenesis in gastrointestinal tumor models [17].

There is compelling evidence linking Wnt signaling to vascularization. In particular, genetic defects in the Wnt receptor, frizzled4, are associated with familial exudative vitreoretinopathy (FEVR), characterized by incomplete retinal neovascularization. Frizzled4^−/−^ mice exhibit leaky vasculature in the retina and cerebellum, and genetic disruption of Wnt2 and frizzled5 results in placental vascularization defects (review [18]). Despite these findings, the mechanism by which Wnt signaling regulates vasculature and which angiogenic factors are linked to Wnt signaling remains unclear. In colon cancer cell lines, Wnt signaling can increase transcription of VEGFA, and elevated levels of VEGFA have been detected in intestinal polyps of APC^min/+^ mice and in human colon cancers relative to matched normal colon tissues [19].

Here, we examined vasculature and potential angiogenic factors in Wnt signaling-induced mammary tumors, and found that SDF1 to be a more important factor than VEGF in facilitating tumor growth. We propose a model in which Wnt1 tumors utilize Gr1^+^CD11b^+^ cells for angiogenesis and growth by producing SDF1.

## Results

### Wnt1 Tumors Are More Vascularized Than Her2 Tumors, and This Correlates with Higher Levels of SDF1, but Not with VEGFA

**Figure 1:**
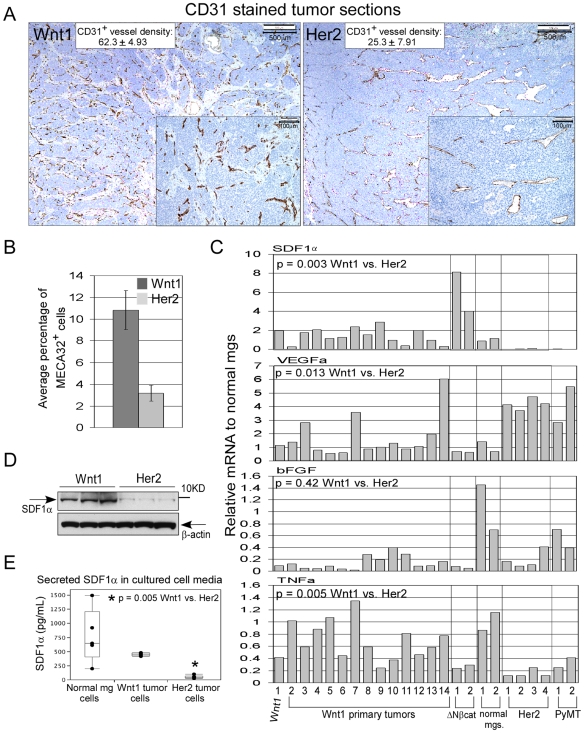
Analysis of Wnt1 tumor vasculature and potential factors that contribute to tumor vasculature. A, immunohistochemical stained Wnt1 and Her2 tumor sections with anti-CD31 antibody (brown) and hematoxylin (blue). The inserts are zoomed in regions of the tumor sections, which were used to score for micro-vessel density. Average numbers of CD31^+^ vessels per 0.45 mm^2^ are shown for each tumor types, and averages were derived from scoring three random sections per tumor, and n = 3 tumors for Wnt1 and Her2. +/− values represent standard deviations. B, quantitation of MECA32^+^ cells in tumors by FACS analysis. Average percentage of MECA32^+^ cells was derived from analysis of five independent Wnt1 (dark bar) and Her2 tumors (light bar), and error bars represent +/− SEM. C, qRT-PCR analysis of indicated mRNA transcript levels in various MMTV tumors and normal mammary glands. Primary Wnt1 tumors are as indicated, and all of the other tumors are from passaged tumors. All reactions were performed in duplicates and relative mRNA level for each gene was calculated by normalizing to average transcript levels of normal mammary glands. D, immunoblot analysis of SDF1 protein immunoprecipitated from lysates of Wnt1 and Her2 tumors, and aliquots of input tumor lysates for immunoprecipitation were blotted for β-actin. E, ELISA analysis of secreted SDF1α protein in media taken from cultured normal mammary gland cells, Wnt1 tumor cells, and Her2 tumor cells. Media were collected from five individually plated tumor or normal mammary cells, and * indicates that lower amount of secreted SDF1α in the Her2 cell media is statistically significant.

We examined the micro-vessel density in Wnt1 and Her2 tumors by immunohistochemical staining of tumor sections with anti-CD31 antibody, a platelet endothelial cell adhesion molecule (PECAM1) marker. Average CD31^+^ vessel counts were 62.3 per 0.45 mm^2^ section for Wnt1 tumors as compared to 25.3 for Her2 tumors (Fig. 1A). Quantitation of MECA32^+^ cells (a pan-endothelial cell marker) in the tumors by fluorescent activating cell sorting (FACS) showed that Wnt1 tumors contained 10.8% MECA32^+^ cells, which was approximately 3-fold higher than the percentage of MECA32^+^ in the Her2 tumors (Fig. 1B). Consistent with the FACS data, Wnt1 tumor sections stained with anti-MECA32 antibody contained a higher proportion of MECA32^+^ cells than in Her2 tumor sections (Fig. S1A).

A number of factors are known to contribute to tumor angiogenesis, including VEGF, basic FGF (bFGF), TNFα, and SDF1. Upon examining these in a panel of primary MMTV tumors and those passaged in mice, we found that the Wnt1 tumors expressed SDF1α and β transcripts at levels at least 7-fold higher than that detected in Her2 or PyMT tumors (Fig. 1C, Fig. S1B; p = 0.003). Higher amounts of SDF1α proteins were also immunoprecipitated from Wnt1 tumor lysates than from Her2 tumor lysates, and higher amounts of secreted SDF1α proteins were detected in the media of cultured Wnt1 tumor cells vs. Her2 tumor cells (Fig. 1D, E). In contrast, VEGFA transcript levels were generally higher in the Her2 and PyMT tumors relative to Wnt1 tumors (Fig. 1C). Expression of bFGF transcript was comparable between Wnt1 and Her2 tumors, and no significant distinction in its expression was noted across the various types of tumors. Slightly higher levels of TNFα transcript were detected in Wnt1 tumors relative to Her2 tumors, although this difference was smaller than that observed for SDF1α. Thus, we focused on determining the role of SDF1 in Wnt-induced tumorigenesis.

### SDF1 Is Produced by Myoepithelial Cells and Stromal Fibroblasts in Wnt1 Tumors

**Figure 2:**
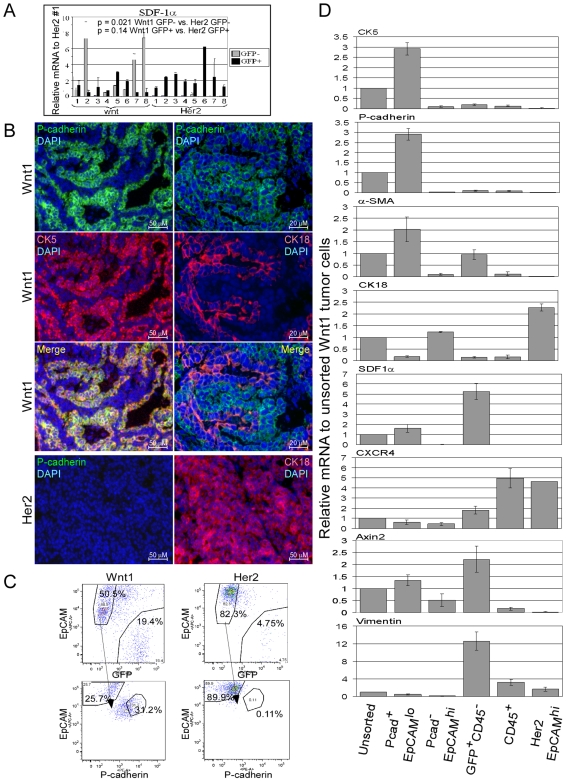
Expression of SDF1 in various cell types from Wnt1 tumors. Dissociated Wnt1 or Her2 tumor cells were injected into the mammary fat pads of EGFPtg/Rag2^−/−^ mice, and isolated GFP^−^ (tumor cells; light bars) and GFP^+^ (stromal cells; black bars) cells were subjected to qRT-PCR analysis of SDF1α expression (A). SDF1α transcript levels were normalized to one of the Her2 GFP^+^ tumor cell sample (lane 1). B, immunofluorescent staining of Wnt1 and Her2 tumor sections with anti-P-cadherin (green), -CK5 (red; left panel), -CK18 antibodies (red; right panel), and DAPI in blue. Merged pictures of P-cadherin staining with CK5 (yellow; left panel) and with CK18 (right panel) are shown. C, FACS gating scheme for isolation of myoepithelial and luminal cells from Wnt1 and Her2 tumors grown in EGFPtg/Rag2^−/−^ mice. EpCAM^+^GFP^−^ cells were further analyzed based on EpCAM and P-cadherin staining. D, qRT-PCR analysis of the indicated mRNA transcripts in the sorted Wnt1 and Her2 tumor cell populations. Relative mRNA transcript levels were normalized to that of the unsorted Wnt1 cell population, and error bars represent standard deviations derived from three independent tumor sorts.

Although SDF1 is typically considered to be secreted by stromal cells, tumor epithelial cells remain a possible source (review [20]). To determine what types of cells in the Wnt1 tumors express SDF1, we injected dissociated Wnt1 tumor cells into the mammary fat pads of immune-deficient Rag2^−/−^mice constitutively expressing an enhanced green fluorescent protein under control of the chicken β-actin promoter (EGFPtg/Rag2^−/−^). The resulting tumor cells can be sorted based on GFP positivity, allowing for the separation of tumor epithelial cells from host stromal cells. Analysis of these isolated cell populations revealed that SDF1α transcript levels were consistently higher in the Wnt1 epithelial cells (GFP^−^) vs. Her2 epithelial cells, whereas SDF1α transcript levels in the Wnt1 stroma cells (GFP^+^) were not significantly higher compared to the Her2 stromal cells (Fig. 2A).

A major distinction between Wnt1 and Her2 tumor epithelial cells is the presence of myoepithelial cells in Wnt1 tumors, which could account for the expression of SDF1. This would be consistent with the reported expression of SDF1 in human mammary myoepithelial cells [21] and with our observed correlation between SDF1α expression and the presence of myoepithelial cell marker transcripts (Fig. S2A). Therefore, we sought to separate the tumor myoepithelial and luminal cell populations to analyze them for expression of SDF1. Although isolation of myoepithelial and luminal cells from human mammary tissues has been described, the cell surface markers (CD10/CALLA for myoepithelial cells and BerEP4 for luminal cells; [21], [22]) used in those isolations are not suitable for mouse cells. Thus, mouse mammary myoepithelial and luminal cells have not been isolated. We found that P-cadherin is a suitable myoepithelial marker for isolation of mouse myoepithelial cells [23]. P-cadherin staining overlapped with that of CK5, a known myoepithelial cell marker, but not with CK18, a luminal cell marker (Fig. 2B). Also, P-cadherin^+^ cells were not found in Her2 tumors, and all of the Her2 tumor cells were CK18^+^.

To isolate myoepithelial cells, tumors were grown in EGFPtg/Rag2^−/−^ mice. Tumor cells were dissociated, incubated with anti-P-cadherin, -EpCAM, and -CD45 antibodies, and sorted by FACS. Hematopoietic cells were first excluded by gating on CD45^−^ populations. Then, the GFP^+^CD45^−^ host cells were gated and separated from the tumor epithelial cells. The remaining GFP^−^EpCAM^+^ cells were further gated based on P-cadherin and EpCAM signals (Fig. 2C).

Upon examining the sorted cells by immunocytochemistry, the P-cadherin^+^EpCAM^lo^ cell fraction was enriched for CK5^+^ cells and depleted for CK18^+^ cells (Fig. S2B). In contrast, P-cadherin^−^EpCAM^hi^ cell fraction was enriched for CK18^+^ cells and depleted for CK5^+^ cells. Consistent with immunocytochemical results, high levels of myoepithelial cell marker transcripts (CK5, P-cadherin, and α-SMA) were found in P-cadherin^+^EpCAM^lo^ cells and high levels of luminal cell transcripts (CK18 and mucin-1) were found in P-cadherin^−^EpCAM^hi^ cells (Fig. 2D, data not shown).

Once myoepithelial and luminal cells were separated, we determined that SDF1α transcript was found only in the myoepithelial cells of Wnt1 tumors, but not in the luminal cells (Fig. 2D). Her2 tumors only contained luminal cells (P-cadherin^−^EpCAM^hi^), which did not express SDF1α. We noted that GFP^+^CD45^−^ cells also expressed SDF1α, and these cells appear to be a fibroblast-enriched population based on their expression of fibroblast markers, vimentin and FSP1 (Fig. 2D, Fig. S2B, data not shown). We also detected much higher levels of secreted SDF1α protein in the media of cultured myoepithelial cells and fibroblasts relative to media of luminal cells (Fig. S2C). Thus, higher levels of SDF1 in the Wnt1 tumors relative to Her2 tumors are due to the presence of myoepithelial cells and higher stromal fibroblasts content.

### SDF1 Expression Is Not Induced by Wnt Signaling

**Figure 3:**
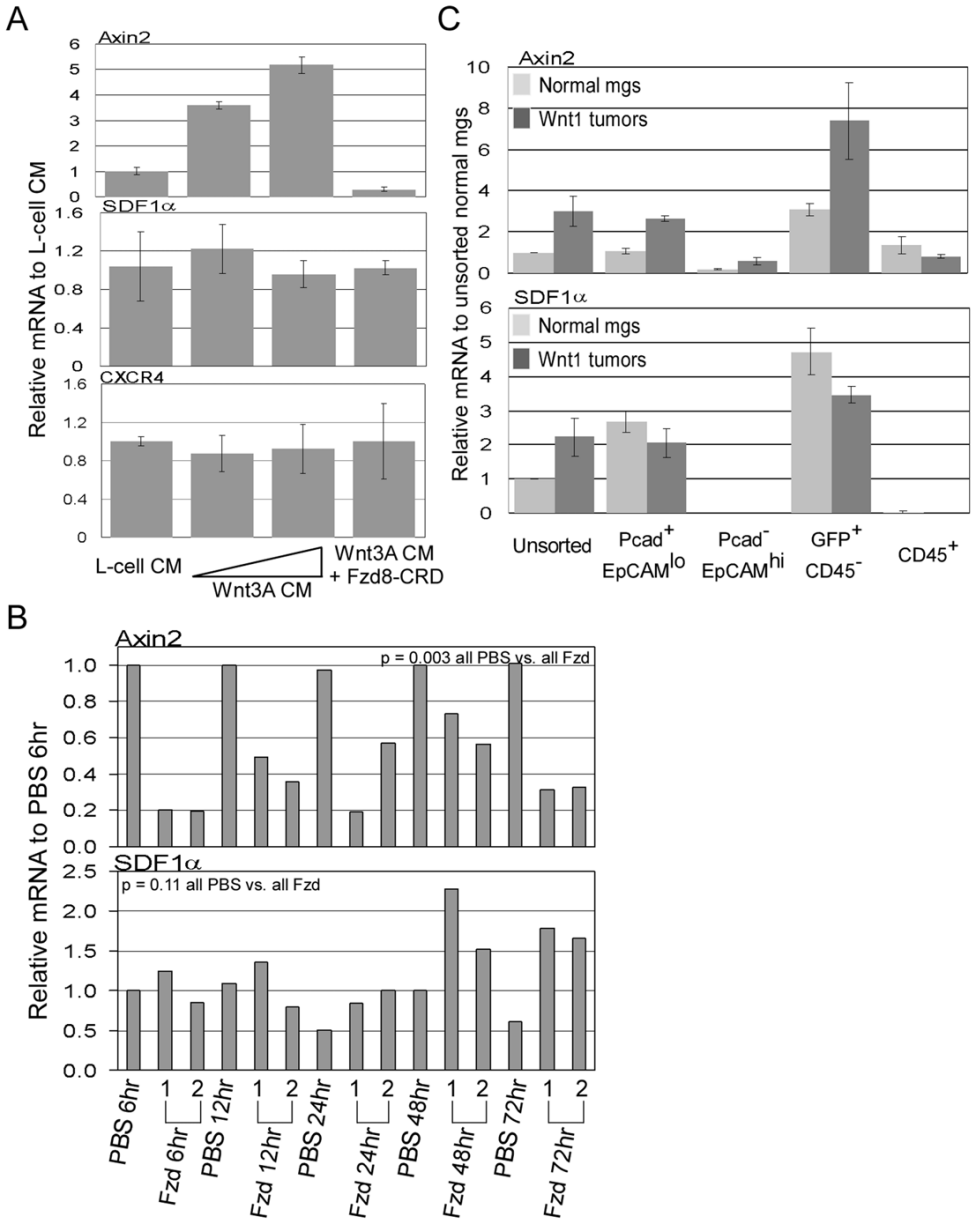
Determination of whether SDF1 is a Wnt signaling responsive gene. A, qRT-PCR analysis of axin2, SDF1α, and CXCR4 transcript levels in normal mammary epithelial cells cultured in Wnt3A conditioned media. Triplicate plated cells were cultured in control L-cell media∶mammary epithelial cell culture media (1∶1 dilution), in Wnt3A conditioned media (1∶2 or 1∶1 dilution), and in Wnt3A conditioned media (1∶1) + Fzd8-CRD (10 µg/mL). Relative transcript levels were normalized to levels in L-cell media. Error bars represent standard deviations derived from triplicate plated cells. B, qRT-PCR analysis of axin2 and SDF1α transcript levels in Wnt1 tumors taken from tumor-bearing mice that were treated with Fzd8-CRD at various hours of post injection. Each time points contain samples from two independently FzD8-CRD treated mice numbered 1 and 2. Relative transcript levels were normalized to the level in the 6 hour PBS-treated tumor sample. **C**, qRT-PCR analysis of axin2 and SDF1α transcript levels in the sorted cell populations from Wnt1 tumors (dark bars) or from normal mammary glands (light bars). Cell populations were isolated from three independent Wnt1 tumors or from three independent pools of normal mammary glands (pools of glands from ∼20 female C57BL6 mice). Error bars represent standard deviations derived from the three independent tumors or pools of mammary gland sorts.

The correlation between the expression of SDF1α and axin2, a known Wnt signaling responsive gene, implies that SDF1 is also a Wnt signaling responsive gene (Fig. 2D). To test this, we cultured primary mammary cells from female C57BL6 mice in conditioned media from Wnt3A-producing L-cells or in L-cell control media. With increasing amount of Wnt3A conditioned media, expression of axin2 transcript increased to a 5-fold maximum relative to control. Also, the increase in axin2 transcript levels was completely inhibited by FzD8-CRD, a soluble Wnt ligand inhibitor (Fig. 3A; [24]). In contrast, SDF1α and CXCR4 transcript levels remained unchanged in cells cultured in Wnt3A conditioned media relative to L-cell media, and were not decreased upon addition of FzD8-CRD. To test whether suppression of Wnt signaling *in vivo* has an effect on SDF1α mRNA transcript, Wnt1 tumor-bearing mice were injected with FzD8-CRD. Axin2 transcript in tumors taken from FzD8-CRD treated mice decreased by ∼3-fold as early as 6 hour post-treatment and remained depressed throughout the time course relative to tumors from PBS treated mice. However, no concomitant decrease in SDF1α transcript levels was observed following FzD8-CRD treatment (Fig. 3B).

Our results indicate that the elevated levels of SDF1 in Wnt1 tumors are not due to a direct activation of this gene by Wnt signaling. Since levels of SDF1α transcript in normal mammary glands and secreted SDF1α in the media of cultured normal mammary cells were not significantly different from that of Wnt1 tumors (Fig. 1C, E), SDF1 expression in the tumors could simply be attributed to the presence of myoepithelial cells and fibroblasts that normally express SDF1. To test this, myoepithelial cells and fibroblasts were isolated from normal mammary glands and Wnt1 tumors. The level of SDF1α transcript was not different between normal myoepithelial cells and fibroblasts and their corresponding cell fractions from Wnt1 tumors (Fig. 3C). In contrast, axin2 transcript levels were 2.4 to 2.6-fold higher in myoepithelial cells and fibroblasts isolated from Wnt1 tumors relative to their corresponding cells from normal mammary glands.

### Inhibition of Wnt Signaling-Induced Tumor Growth by Neutralization of SDF1

**Figure 4:**
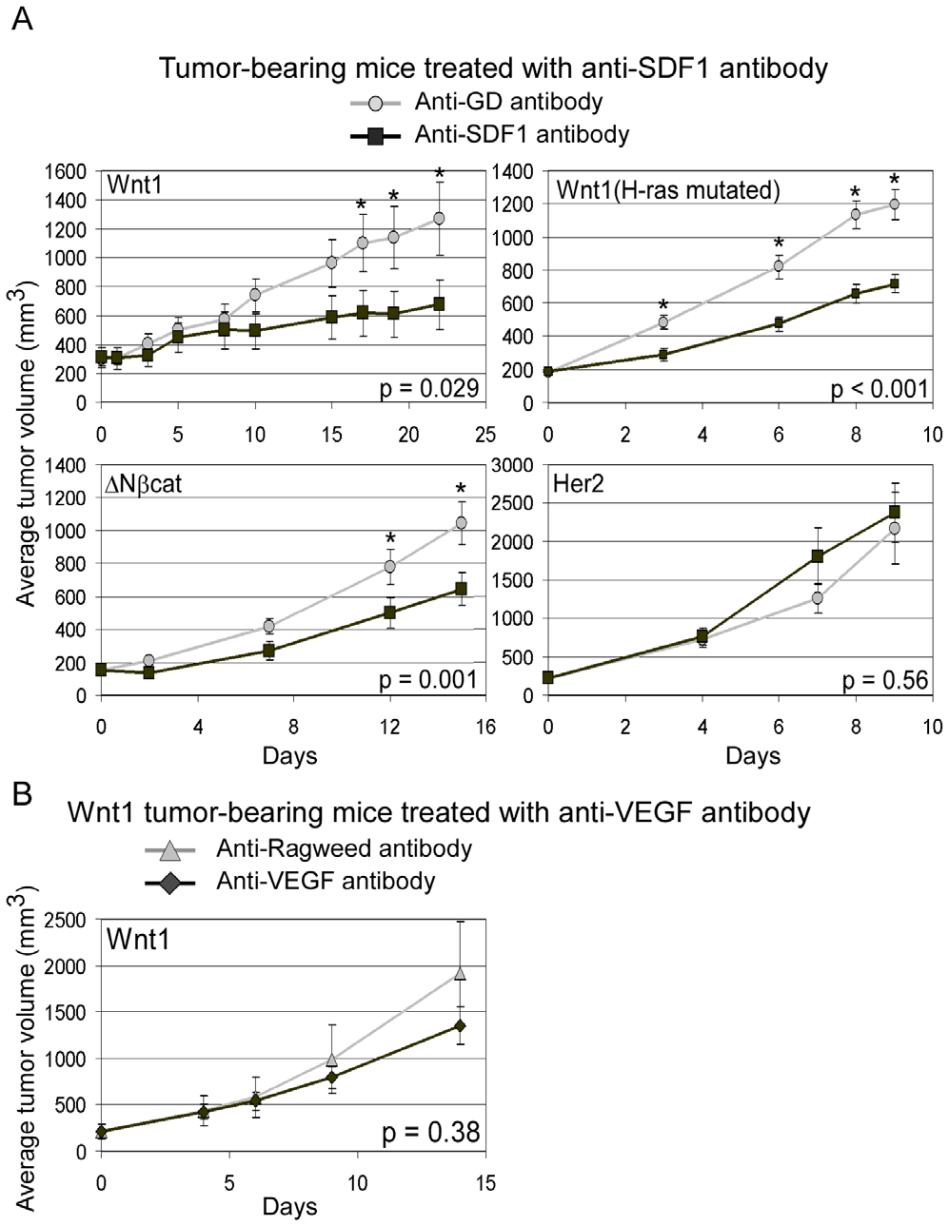
MMTV tumor-bearing mice treated with anti-SDF1 and anti-VEGF antibodies. Dissociated MMTV tumor cells were injected into nude mice, and tumor-bearing mice were injected with anti-SDF1 (circle) or control anti-GD (square) antibodies (A) and injected with anti-VEGF (diamond) or control anti-Ragweed antibodies (triangle; B). Average tumor volume was calculated from n = 10 treated mice for anti-SDF1 treatments and n = 5 for anti-VEGF treatment. Error bars represent +/− SEM. * indicates average tumor volumes that are statistically different between anti-GD and anti-SDF1 treatment groups (p<0.05).

Since MMTV-Wnt1 tumors expressed significantly higher levels of SDF1 than the other MMTV tumors (Fig. 1C–E), we asked whether SDF1 was functionally relevant to tumorigenesis. To test this, dissociated tumor cells were injected into nude mice, and tumor-bearing mice were treated with functional perturbing anti-SDF1 antibody or with control anti-HSV Glycoprotein D (GD) antibody three times per week. Following treatments, anti-SDF1 antibody significantly inhibited Wnt1 tumor growth, and the average tumor size of control anti-GD treated group (n = 10) was 1,266 mm^3^ where as anti-SDF1 treated group was 674 mm^3^ (p = 0.029; Fig. 4A). We also repeated this experiment with a different MMTV-Wnt1 tumor line that harbors a spontaneous H-ras mutation. Although this tumor grew much faster than the previous Wnt1 tumor without H-ras mutation, a similar inhibition of tumor growth with anti-SDF1 antibody was seen (1,196 mm^3^ for anti-GD group vs. 716 mm^3^ for anti-SDF1 group; p<0.001). Since MMTV-ΔNβcat tumors also expressed high levels of SDF1, we treated ΔNβcat tumor-bearing mice with anti-SDF1 antibody and again saw a significant inhibition in average tumor growth (anti-GD group: 1,043 mm^3^ vs. anti-SDF1 group: 644 mm3; p = 0.001). Finally, we tested the Her2 tumors, which express very low levels of SDF1 and found that anti-SDF1 antibody did not inhibit their growth (anti-GD group: 2,170 mm^3^ vs. anti-SDF1 group: 2,300 mm^3^; p = 0.56). These results demonstrate that SDF1, which can impact tumor angiogenesis, significantly contributes to the growth of Wnt1 and ΔNβcat tumors. Nevertheless, VEGF, previously reported to be a Wnt signaling responsive gene, might also play a significant role in angiogenesis and growth of the Wnt1 tumors. To test this, Wnt1 tumor-bearing mice were treated with anti-VEGF antibody (blocks both VEGFA and VEGFB [25]) or with anti-Ragweed control antibody three times per week. After the treatment, the average tumor size for the anti-VEGF treated group was slightly smaller than the anti-Ragweed treated group (1,354 mm^3^ vs. 1,910 mm^3^), but this difference was not statistically significant (Fig. 4B; p = 0.38).

### Anti-SDF1 Antibody Treatment Reduces Gr1^+^CD11b^+^ Cells and Endothelial Cells in the Wnt1 Tumors

**Figure 5:**
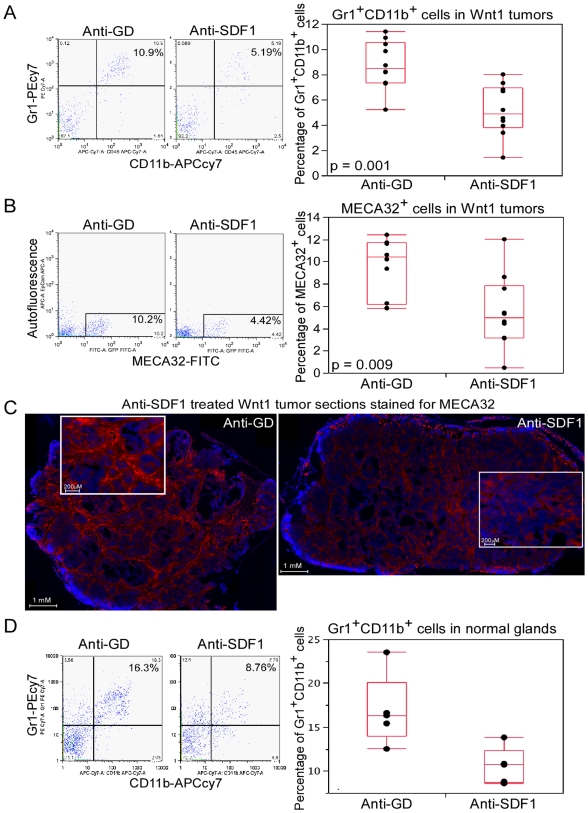
Assessment of BMDC contributions and endothelial cell content in Wnt1 tumors and normal mammary glands following anti-SDF1 treatment. FACS profiles and box plots of percentage of Gr1^+^CD11b^+^ cells (A) and MECA32^+^ cells (B) in Wnt1 tumors (n = 10) following four days of anti-SDF1 treatment. C, immunofluorescent staining of Wnt1 tumor sections with anti-MECA32 antibody (red) and DAPI (blue) following four days of anti-SDF1 treatment. Entire tumor section was assembled from ∼100 images taken by fluorescent microscope with a 10X objective, and inserts are zoom in depictions of that particular sections. D, FACS profiles and box plots of percentage of Gr1^+^CD11b^+^ cells in normal virgin mammary glands following four days of anti-SDF1 treatment.

The impact of anti-SDF1 treatment on Wnt1 tumor growth prompted us to examine the mechanism by which SDF1 contributes to Wnt1 tumor growth. It is known that SDF1 can recruit BMDCs to facilitate tumor angiogenesis. Therefore, we tested whether anti-SDF1 treatment can affect the amount of BMDCs in the Wnt1 tumors. Among the various BMDCs, including endothelial progenitor cells (CD34^+^VEGFR2^+^ and CD31^+^Sca1^+^), macrophages (F4/80^+^), and myeloid progenitor cells (Gr1^+^CD11b^+^), the Gr1^+^CD11b^+^ cells underwent the most consistent change following anti-SDF1 treatment (Fig. 5A, Fig. S3A–C). At day 4 following anti-SDF1 treatments, the median percentage of Gr1^+^CD11b^+^ cells in the anti-SDF1 treated tumors was 4.86 and 8.47 for the anti-GD control treated tumors (p = 0.001). Consistent with the proposal that Gr1^+^CD11b^+^ cells contribute to tumor angiogenesis, the percentage of endothelial cells (MECA32^+^) was also decreased following anti-SDF1 treatment (anti-SDF1 group: 4.95 vs. anti-GD group: 10.4; p = 0.009; Fig. 5B). Immunohistochemical staining of Wnt1 tumor sections showed that regions lacking MECA32^+^ cells were more abundant and stained vessels were much slimmer in the anti-SDF1 treated tumors relative to control (Fig. 5C). Since similar levels of SDF1 expression was found in normal mammary glands relative to Wnt1 tumors, we also determined the effect of anti-SDF1 treatment on the amount of Gr1^+^CD11b^+^ cells residing in the normal mammary glands. Normal mammary glands contained ∼16.3% of Gr1^+^CD11b^+^ cells, and these cells were reduced to 10.7% following anti-SDF1 treatment (p = 0.022; Fig. 5D), suggesting that both Wnt1 tumors and normal mammary glands utilize SDF1 to harbor these Gr1^+^CD11b^+^ cells.

### Depletion of Total Gr1^+^ Cells in Mice Inhibits Wnt1 Tumor Growth

**Figure 6:**
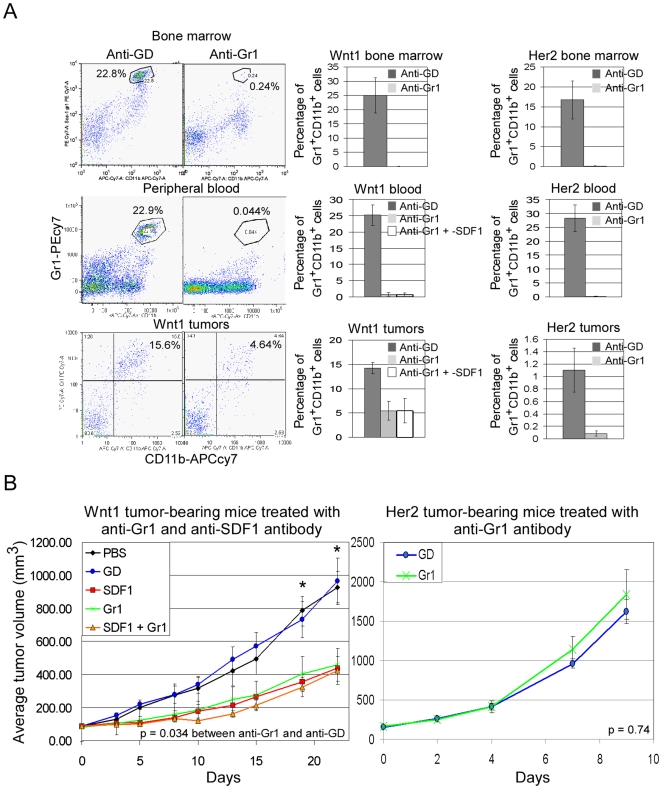
Tumor-bearing mice treated with anti-Gr1 and anti-SDF1 antibody. A, FACS profiles and histogram plots of percentage of Gr1^+^CD11b^+^ cells in the bone marrow, peripheral blood, and tumors of Wnt1 and Her2 tumor-bearing mice treated with anti-GD (dark bars), anti-Gr1 (light bars), or anti-Gr1 and -SDF1(white bars) antibody. Error bars represent standard deviations derived from analysis of three independently treated mice. B, average tumor volumes of Wnt1 (left panel) or Her2 (right panel) tumor-bearing mice treated with controls, PBS (black) and anti-GD (blue), and with anti-SDF1 (red), -Gr1 (green) and both -SDF1 plus -Gr1 (orange) antibodies. Average tumor volume was determined from n = 5 mice per treatment group, and error bars represent +/− SEM. * indicates average tumor volumes that are statistically different between anti-GD and anti-Gr1 treatment groups (p<0.05).

To test whether Gr1^+^ cells are important for Wnt1 tumor growth, the Gr1^+^ cells can be depleted *in vivo* by administration of anti-Gr1 antibody [26]. Wnt1 and Her2 tumor-bearing mice were injected with anti-Gr1 (twice per week), anti-SDF1 (three times per week), and a combination of both antibodies. Virtually all of the Gr1^+^ cells (mostly neutrophils) and the Gr1^+^CD11b^+^ double positive cells (neutrophils and macrophages lineage) from bone marrow and peripheral blood of tumor-bearing mice were depleted upon treatment with anti-Gr1 antibody (Fig. 6A). Also, the Gr1^+^CD11b^+^ cells in the tumors were significantly reduced with anti-Gr1 treatment.

Following antibody treatments, average Wnt1 tumor size in the anti-Gr1 group was inhibited by 53% relative to control anti-GD treated group (Fig. 6B; p = 0.034), which was similar to the levels of inhibition by anti-SDF1. However, anti-SDF1 treatment did not add to the tumor inhibition observed in animals already depleted of Gr1^+^ cells. These data suggest that Gr1^+^ cells are important for Wnt1 tumor growth, and that SDF1 is responsible for the involvement of these cells with Wnt1 tumors. Consistent with SDF1 involving Gr1 cells, growth of Her2 tumors, which express very low levels of SDF1, was not affected by anti-Gr1 depletion (Fig. 6B).

## Discussion

### SDF1 Is an Important Factor for the Growth of Wnt Signaling-Induced Mammary Tumors

It has been observed that MMTV-Wnt1 tumors frequently contain blood-filled cysts and lymphocytic infiltrates [3], [6]. However, tumor vasculature and factors that can contribute to vascularization have not been clearly described for the Wnt1 tumors. Here, we report that Wnt1 tumors are more vascularized than Her2 tumors, and SDF1 is a key factor in facilitating Wnt1 tumor growth and angiogenesis (Fig. 1 and 4).

We showed that Gr1^+^CD11b^+^ cells are important for Wnt1-induced tumorigenesis, and their presence in the Wnt1 tumors depends on SDF1 function. We detected a decrease in Gr1^+^CD11b^+^ cells in the Wnt1 tumors following anti-SDF1 treatment (Fig. 5A), consistent with the reported ability of SDF1 to attract these cells [15]. Our depletion of Gr1^+^ cells in Wnt1 tumor-bearing mice gave similar but no additional inhibition on Wnt1 tumor growth when combined with anti-SDF1, suggesting that SDF1 and Gr1^+^ cells have overlapping mechanisms in facilitating tumor growth. Also, in accord with the reliance of Wnt1 tumors upon Gr1^+^CD11b^+^ cells for their growth, Wnt1 tumors are more resistant to anti-VEGF treatment than anti-SDF1 treatment, a property that was shown to be attributed to these Gr1^+^CD11b^+^ cells (Fig. 4B, [27]).

Gr1^+^CD11b^+^cells were first described as myeloid immune suppressor cells that can inhibit maturation of antigen-presenting dendritic cells, presumably allowing tumors to escape immune surveillance [28]–[30]. However, we can exclude this mechanism as our allograft tumors were grown in immune-deficient mice. More recent findings support an alternative role for these cells in contributing to tumor angiogenesis. Gr1^+^CD11b^+^ cells are incorporated into CD31^+^ blood vessels in tumors, where they produce high levels of MMP9 which is required for newly synthesized vessels [8], [31]. Gr1^+^CD11b^+^ cells also express Bv8 to enhance mobilization and facilitate tumor angiogenesis [32]. Thus, Gr1^+^CD11b^+^ cells might contribute to the vasculature in Wnt1 tumors, and we showed that a reduction in these cells correlated with a decrease in endothelial cells in the Wnt1 tumors following anti-SDF1 treatment (Fig. 5). We also found that Gr1^+^CD11b^+^ cells were significantly elevated in the peripheral blood of tumor-bearing mice following anti-SDF1 treatment (Fig. S3D). We believe that inhibiting SDF1 function prevents retention of Gr1^+^CD11b^+^ cells in tissues (including tumors) that normally express high levels of SDF1. Thus, anti-SDF1 treatment releases Gr1^+^CD11b^+^ cells into blood circulation, precluding these cells from participating in tumor vasculogenesis and tumorigenesis. This interpretation agrees with the finding that CXCR4 antagonist, AMD3100, treatment induces rapid mobilization of murine and human hematopoietic stem/progenitor cells [33].

It is also possible that SDF1 directly stimulates Wnt1 tumor cells, as SDF1 has been shown to increase proliferation of human breast cancer cell lines in *in vitro* cultures [21], [34]. Although we did observe a slight decrease in Ki67^+^ cells in Wnt1 tumors following anti-SDF1 treatment (data not shown), this could have been an indirect effect resulting from a reduction in Gr1^+^CD11b^+^ cells and/or angiogenesis. Indeed the effect of anti-SDF appeared to be mediated by Gr1^+^ cells because no further inhibition by anti-SDF1 was observed following their depletion. Moreover, in vitro culturing of Wnt1 tumor cells in media containing purified SDF1 or anti-SDF1 antibody did not show significant differences in total cell viability (Fig. S4).

### Wnt1 Tumors Use Factors Produced by Normal Mammary Developmental Process for Their Growth

Elevated SDF1 expression has been described in a number of tumorigenic conditions relative to normal settings. First, higher SDF1 expression is found in tumor enhancing cancer-associated fibroblast relative to patient matched non-cancerous mammary fibroblasts [7]. Second, SDF1 expression is induced under hypoxic conditions in a glioblastoma tumor model system through HIF1α, suggesting that SDF1 expression is increased in rapidly growing tumors deprived of oxygen supply [35]. Finally, higher levels of SDF1 expression are found in myoepithelial cells and myofibroblasts of human ductal carcinoma in situ and invasive breast cancers relative to the same cell fractions in normal mammary glands [21]. Although we found high levels of SDF1 in Wnt signaling-induced tumors, the expression was comparable to that of normal mouse mammary glands, which is consistent with previous findings [14]. Further fractionation revealed that the levels of SDF1 expression in the myoepithelial cells and fibroblasts isolated from normal mammary glands are comparable to the levels found in the counterparts isolated from Wnt1 tumors (Fig. 3C). Thus, high levels of SDF1 expression in the Wnt1 tumors are due to the presence of myoepithelial cells and/or stromal fibroblasts, and unlikely associated with elevated Wnt signaling.

It is also likely that the role of SDF1 in Wnt1-induced tumorigenesis is similar to that in normal mammary gland development. We did detect a decrease in Gr1^+^CD11b^+^ cells in both the Wnt1 tumors and in normal mammary glands following anti-SDF1 treatment (Fig. 5A, D). Moreover, recruitment of other BMDCs such as macrophages and eosinophiles to the mammary glands is a critical part of normal developmental process, as these cells typically reside at the leading edge of developing mammary terminal end buds [36]. The absence of these BDMCs results in retardation of mammary ductal branching. Thus, Wnt tumors may not depend on production of de novo factors for their growth; but rather co-opt the existing resources available to them.

In conclusion, we demonstrate that SDF1 is a key factor in Wnt signaling-induced mammary gland tumorigenesis. The source of SDF1 is from myoepithelial cells and stromal fibroblasts of Wnt signaling-induced tumors, and SDF1 supports tumorigenesis by involving Gr1^+^CD11b^+^ cells. We propose that anti-SDF1 may be an effective therapeutic strategy for targeting mammary tumors with abundant amounts of myoepithelial cells and/or stromal cells expressing SDF1.

## Materials and Methods

### Mice and Allograft Tumors

To generate immune-deficient mice constitutively expressing GFP (EGFPtg/Rag2^−/−^), EGFP transgenic mice (C57BL/6-Tg (ACTB-EGFP) 1Osb/J, Jackson Laboratory) were crossed with mice with a genetic disruption of Rag2 gene (B6.SJL (129S6)-*Ptprc^a^*/BoyCrTac-*Rag2^tm1Fwa^*, Taconic). Athymic NCR-nude mice (Taconic) were used for MMTV tumor transplants and antibody treatments. Wnt1 tumors used for transplantations were from MMTV-Wnt1 transgenic mice that were passaged in mammary fat pads of C57/BL6 mice [24]. Her2 tumors were from MMTV.f.huHER2 mice and passaged in FBV/N mice as described in [37]. ΔNβcat tumors were obtained from Dr. Cowin (NYU), and tumors were passaged in nude mice. All experimental protocols using mice were approved by the Institutional Animal Care and Use Committee at Genentech Inc.

### Immunohistochemistry and Scoring of Micro-Vessel Density

Tumors were fixed in 10% formalin, embedded in paraffin for histological sectioning, and sections were subjected to antigen retrieval following the protocol described from the Rosen laboratory (http://www.bcm.edu/rosenlab/protocols/generalIF.pdf). Tumor sections were blocked by MOM (Vector Laboratories), and incubated with primary antibodies, anti-CD31 (Abcam), -MECA32 (BD Biosciences), -P-cadherin (R&D Systems, goat polyclonal), -cytokeratin 5 (CK5; Abcam) and -cytokeratin 18 (CK18; Abcam, mouse monoclonal). For immunohistochemical staining of sections, HRP-conjugated secondary antibody was used followed by hematoxylin counterstaining. Scoring of micro-vessel density was done by using a modified method described in [38]. Tumor sections were scanned at a low magnification (40x) to identify areas with the highest density of CD31^+^ blood vessels. Three random areas with the highest vessel density were counted at a higher magnification (200x; ∼0.45 mm^3^ section area), and only structures with clear identifiable lumen were scored.

For immunofluorescent staining, Alexa488- or Alexa555-conjugated secondary antibody was used followed by mounting with Prolong Gold (Invitrogen) containing DAPI dye. Fluorescent pictures were taken by Axiovert fluorescent microscope (Zeiss) and images were processed with Axiovision and Adobe Photoshop software.

### Quantitative Real-Time PCR (qRT-PCR)

RNeasy miniprep kit (Qiagen) was used to isolate total RNA from mammary tissues, tumors, and cells. 50 ng of RNA was mixed in a 25 uL qRT-PCR master mix (Applied Biosystems Inc; ABI), and reactions were carried out in a 7500 Real-time PCR system. Relative transcript level for each gene was determined by using 2^−ΔΔCt^ method and normalized to housekeeping RPL19 transcript level. Taqman^TM^ primers used, RPL19: 5′- GCGCATCCTCAT GGAGCACA, 3′- GGTCAGCCAGGAGCTTCTTG, probe -CACAAGCTGAAGGCAGACAAGGCCC; Mouse Axin2: 5′- GTGAGCTGGTTG TCACCTACTT, 3′- AAGCTTTGAGCCTTCAGCAT, probe - TTCTGTGGAGAA GAAATTCCATACAGGAGG; Mouse SDF1α: 5′-CATCCATCCATCCATCCA, 3′- TTCAGGGTCATGGAGACAGT, probe -TCATCGCCATGTGTCCGCAAG. All other primers and probes were purchased from ABI.

### Immunoprecipitation of SDF1 Protein from MMTV Tumors

Tumor tissues were homogenized in lysis buffer (1 g of tissue per 10 mL of lysis buffer; Invitrogen) using Qiagen tissue homogenization system. Total protein concentration was determined by BCA assay (Pierce), and 50 mg of protein lysate was used in the immunoprecipitation with 100 µg of anti-SDF1 antibody (R&D Systems). Immunoprecipitated protein was subjected to immunoblot analysis using anti-SDF1 antibody (Cell Signaling Technology). Aliquots of input lysates for immunoprecipitation were also subjected to immunoblot analysis by using anti-β-actin antibody as a loading control.

### ELISA Analysis of Cultured Wnt1 Tumor Cells, Her2 Tumor Cells and Normal Mammary Gland Cells

Normal mammary glands (from 4–7 month-old C57BL/6 mice), Wnt1, and Her2 tumors were dissected, and cells were disaggregated as described below. Cells were plated on 12 well collagen I-coated plates (BD Bioscience) in 300 µL of mouse mammary epithelial cell cultured media (Stemcell Technologies). Once cells were confluent, media were collected and total protein in media was determined by BCA assay. Media containing equal amount of protein (200 µg) were used in ELISA assay by using Mouse CXCL12/SDF1α Quantikine ELISA Kit (R&D Systems). Colorimetric values were measured by microplate reader (SpectraMax 190) at 450 nm, and nonspecific readings at 540 nm were subtracted from values measured at 450 nm.

### Isolation of Myoepithelial and Luminal Cells

When Wnt1 or Her2 tumors grown in EGFPtg/Rag2^−/−^ mice reached ∼500–1000 mm^3^, tumors were dissected from mice, minced into tiny pieces, and incubated in digestion media I (F12 media containing 4 mg/mL of collagenase I (sigma), 150 µg/mL of hyaluronidase (sigma), 5% fetal bovin serum) for 90 minutes at 37°C with rocking. Red blood cells were lysed in hypotonic solution. Semi-dissociated tumor cells were further digested to single cells by incubation in digestion media II (F12 media containing 4 mg/mL of collagenase I, 150 µg/mL of hyaluronidase, 5 mg/mL of dispase type II (Roche), 50 U/mL of DNase I (Roche)) for 5 minutes at 37°C, then followed by incubation in trypsin 0.25% +EDTA (Invitrogen) + 10 mM CaCl_2_ for 5 minutes at 37°C. Trypsin was inactivated by washing cells twice with RPMI + 10% FBS. Single cells were resuspended in washing solution (PBS+1% BSA) and filtered through a 40 µM mesh. Normal #4 and #5 mammary glands were dissected from twenty 4–7 month-old female C57BL/6 mice, chopped into small pieces, and digested similarly as tumor cells. Dissociated cells were incubated with P-cadherin antibody (R&D Systems) for 30 minutes at 4°C with shaking. Cells were washed and incubated with secondary PE-conjugated anti-goat IgG, APC-conjugated EpCAM (Biolegend), and APCcy7-conjugated CD45 (BD Bioscience) antibodies for 30 minutes at 4°C. Following staining, cells were resuspended in propidium iodide (5 µg/mL) and sorted by FACSAria^TM^ cell sorter. FACS data were analyzed by FlowJo software (Tree Star Inc.).

### Wnt3A Conditioned Media Treatment of Primary Normal Mammary Gland Cells

Dissociated primary mammary cells from C57BL/6 mice were plated on collagen I-coated plates (BD Bioscience) in mouse mammary epithelial cell cultured media. Following two days of culture, cells were incubated with Wnt3A conditioned media (from Wnt3A producing L-cells) or with control media (from L-cells) for another 24 hours at which cells were collected and total RNA was extracted. Conditioned media was diluted at various ratios with mammary cell culture media as indicated.

### *In Vitro* Treatment of Wnt1 Tumor Cells with Purified SDF1 and Anti-SDF1 Antibody

Dissociated Wnt1 tumor cells were plated on 24 well collagen I-coated plates (BD Bioscience) in 200 µL of mouse mammary epithelial cell cultured media containing purified SDF1 (R&D Systems), anti-GD, or anti-SDF1 antibody. Total cell viability was determined by using CellTiter-Glo Luminescent Cell Viability Assay (Promega). Luminescent signal was measured by Perkin Elmer Envision 2102 multi-label plate reader.

### Antibody Treatment of MMTV Tumor-Bearing Mice

Dissociated tumor cells (0.5×10^6^) were resuspended in HBSS:matrigel (1∶1) and injected into the #3 mammary fat pads of 8–12 week-old NCR-nude mice. Mice with similar size of tumors (100–200 mm^3^) were selected for the following antibody treatments. Tumor-bearing mice were given intravenous (i.v.) injections of hamster anti-SDF1 antibody (25 mg/kg; raised against recombinant human SDF1; Genentech Inc.) three times per week or with the same dose of hamster anti-HSV Glycoprotein D (GD) control antibody. Anti-SDF1 antibody has been tested to inhibit both SDF1α and β forms and it cross-reacts with mouse SDF1 (data not shown). Nude mice were used for the antibody treatments to avoid immune rejection of anti-SDF1 antibody because it was generated in hamster. For anti-VEGF treatments, mice were given intraperitoneal (i.p.) injections of anti-VEGF antibody (5 mg/kg; [25]) or injections of anti-Ragweed control antibody three times per week. Anti-Gr1 (Ly-6G/C Ly-6G) antibody treatment was done by i.p. injections of mice twice per week (10 mg/kg; BD Bioscience).

### Treatment of Wnt1 Tumor-Bearing Mice with Fzd8-CRD

Fzd8-CRD was purified as described in [24]. When Wnt1 tumors grown in EGFPtg/Rag2^−/−^ mice reached ∼500–1000 mm^3^, tumor-bearing mice were i.p. injected with Fzd8-CRD (10 mg/kg). Tumors were collected at various time points post injection, and total RNA was isolated.

### Statistical Analysis

P values for tumor growth were calculated by Kaplan-Meier Time to Progression method, and p values for two sample comparisons were calculated by two-sided Wilcoxon Rank-Sum test. JMP software was used for statistical calculations.

## Supporting Information

Figure S1 Analysis of Wnt1 tumor vasculature and potential factors that contribute to tumor vasculature. A, immunofluorescent stained Wnt1 and Her2 tumor sections with anti-MECA32 antibody (red) and DAPI (blue). The entire tumor section was assembled from images taken by fluorescent microscope with a 10X objective, and inserts are zoom in depictions of that particular sections. Total MECA32+ pixels were counted and divided by total DAPI pixels to generate the percentage of MECA32+ pixels. B, qRT-PCR analysis of SDF1α (light bars) and β (black bars) mRNA transcripts in Wnt1 and Her2 tumors. All Taqman reactions were performed in duplicate and normalized to housekeeping RPL19 transcript level. Relative mRNA level for each gene was calculated by the 2^−ΔΔCt^ method by normalizing to average transcript levels of first Her2 tumor (#1) sample.
(8.76 MB TIF)

Figure S2 Determination of cells in Wnt1 tumors that produce SDF1. A, qRT-PCR analysis of SDF1α, myoepithelial cell, and luminal cell markers mRNA transcripts in various MMTV tumors. Myoepithelial cell maker transcripts are shown as a combined group: CK5 in stripped bars, P-cadherin in grey bars, and α-SMA in black bars. Primary Wnt1 tumors are as indicated and all of the other tumors are from passaged tumors. Relative mRNA level for each gene was normalized to average transcript levels of normal mammary glands. B, immunocytochemical analysis of the sorted Wnt1 tumor cell populations stained with anti-CK5 (green), -CK18 (red), and -vimentin (orange) antibodies, and DAPI stained nuclei (blue). Immunofluorescent staining was done on sorted cells by spotting sorted cells on glass slides, allowing cells to be dried onto the slides, then fixing cells with 4% paraformaldehyde for 10 minutes at room temperature, and permeabilizing cells with cold methanol for 10 minutes at 4oC. Fixed cells was blocked with MOM (Vector Laboratories), incubated with primary antibodies, followed by incubation with secondary antibodies. Finally, cells were washed and mounted by Prolong Gold (Invitrogen) containing DAPI dye. C, ELISA analysis of secreted SDF1α protein in media taken from cultured myoepithelial cells (Pcad^+^EpCAM^lo^), luminal cells (Pcad^−^EpCAM^hi^), and stromal fibroblasts (GFP+CD45-) sorted from Wnt1 tumors. Error bars represent standard deviations derived from triplicate plated cells.
(10.21 MB TIF)

Figure S3 Assessment of various BMDCs that may contribute to tumor angiogenesis following anti-SDF1 treatment of Wnt1 tumors. FACS profiles and histogram/box plots of endothelial progenitor cells CD34+VEGFR2+ cells (A), CD31+Sca1+ cells (B), and tumor-associated macrophages (F4/80+; C) in the Wnt1 tumors following anti-SDF1 treatment of tumor-bearing mice. Indicated number in the FACS profile represents percentage of cells within the designated gate. P value was calculated by two-sided Wilcoxon Rank-Sum test, and error bars represent standard deviations from three independent FACs analysis. D, FACS profiles and box plots of Gr1+CD11b+ cells in the peripheral blood of Wnt1 tumor-bearing mice (n = 10) following anti-SDF1 or control anti-GD treatment.
(9.68 MB TIF)

Figure S4 Wnt1 tumor cells treated with purified SDF1 and anti-SDF1 antibody. Dissociated Wnt1 tumor cells were cultured in media containing control anti-GD antibody (30 µg/mL; blue), SDF1 (100ng/mL; green) or various amounts of anti-SDF1 antibody (30 µg/mL in red or 100 µg/mL in orange). Cell viability was measured everyday following treatments, and average cell viability (RLUs) was calculated from three independently plated cells. Error bars represent standard deviations.
(7.36 MB TIF)
