# Supplementary figures and images for: Stromal Cell-Derived Factor-1/CXCL12 Contributes to MMTV-Wnt1 Tumor Growth Involving Gr1+CD11b+ Cells
Source: PLoS One. 2010 Jan 19;5(1):e8611. doi: 10.1371/journal.pone.0008611 (PMC2801592; doi:10.1371/journal.pone.0008611)

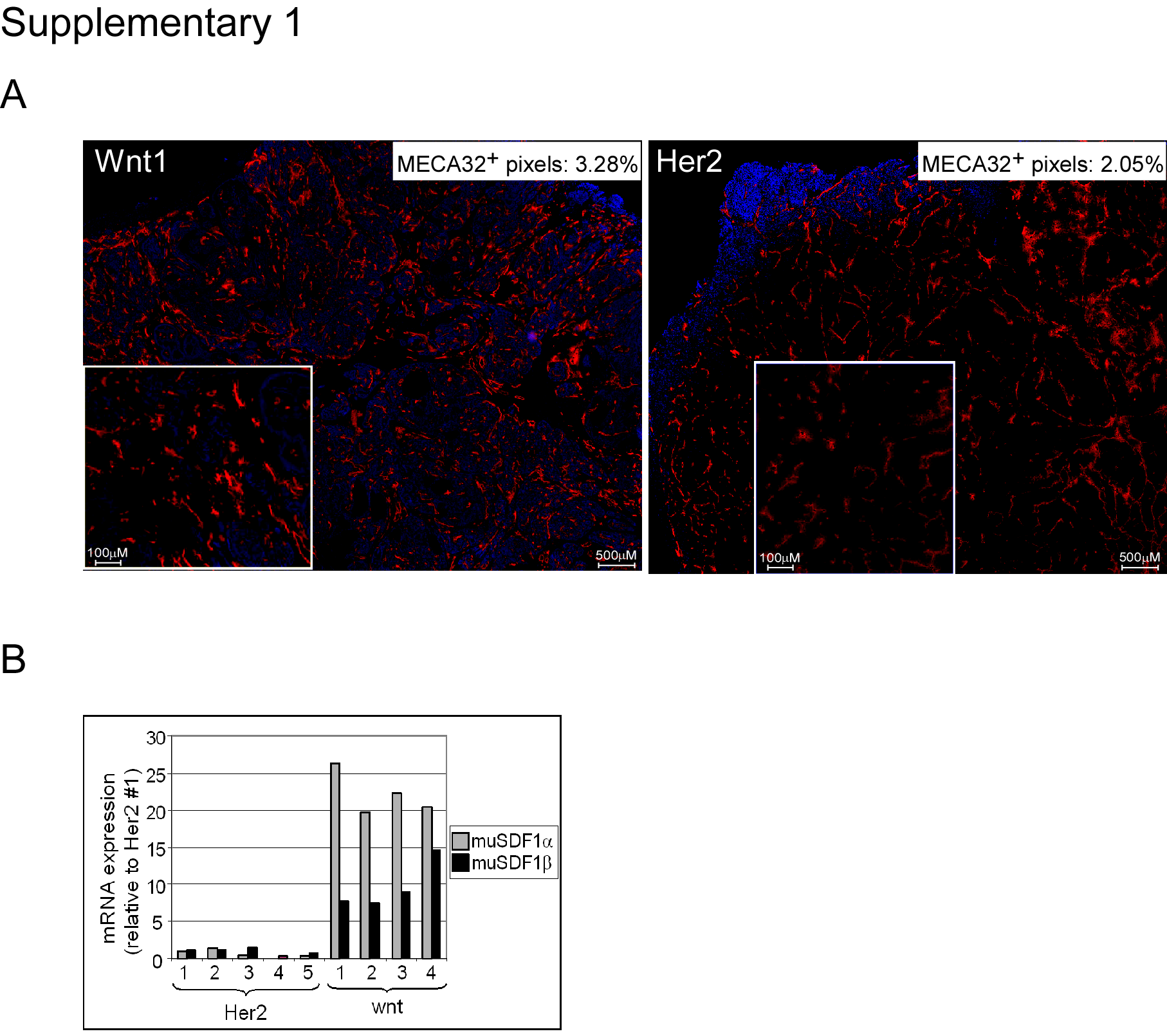

Supplement: Figure S1 — Analysis of Wnt1 tumor vasculature and potential factors that contribute to tumor vasculature. A, immunofluorescent stained Wnt1 and Her2 tumor sections with anti-MECA32 antibody (red) and DAPI (blue). The entire tumor section was assembled from images taken by fluorescent microscope with a 10X objective, and inserts are zoom in depictions of that particular sections. Total MECA32+ pixels were counted and divided by total DAPI pixels to generate the percentage of MECA32+ pixels. B, qRT-PCR analysis of SDF1α (light bars) and β (black bars) mRNA transcripts in Wnt1 and Her2 tumors. All Taqman reactions were performed in duplicate and normalized to housekeeping RPL19 transcript level. Relative mRNA level for each gene was calculated by the 2−ΔΔCt method by normalizing to average transcript levels of first Her2 tumor (#1) sample. (8.76 MB TIF) [file pone.0008611.s001.tif]

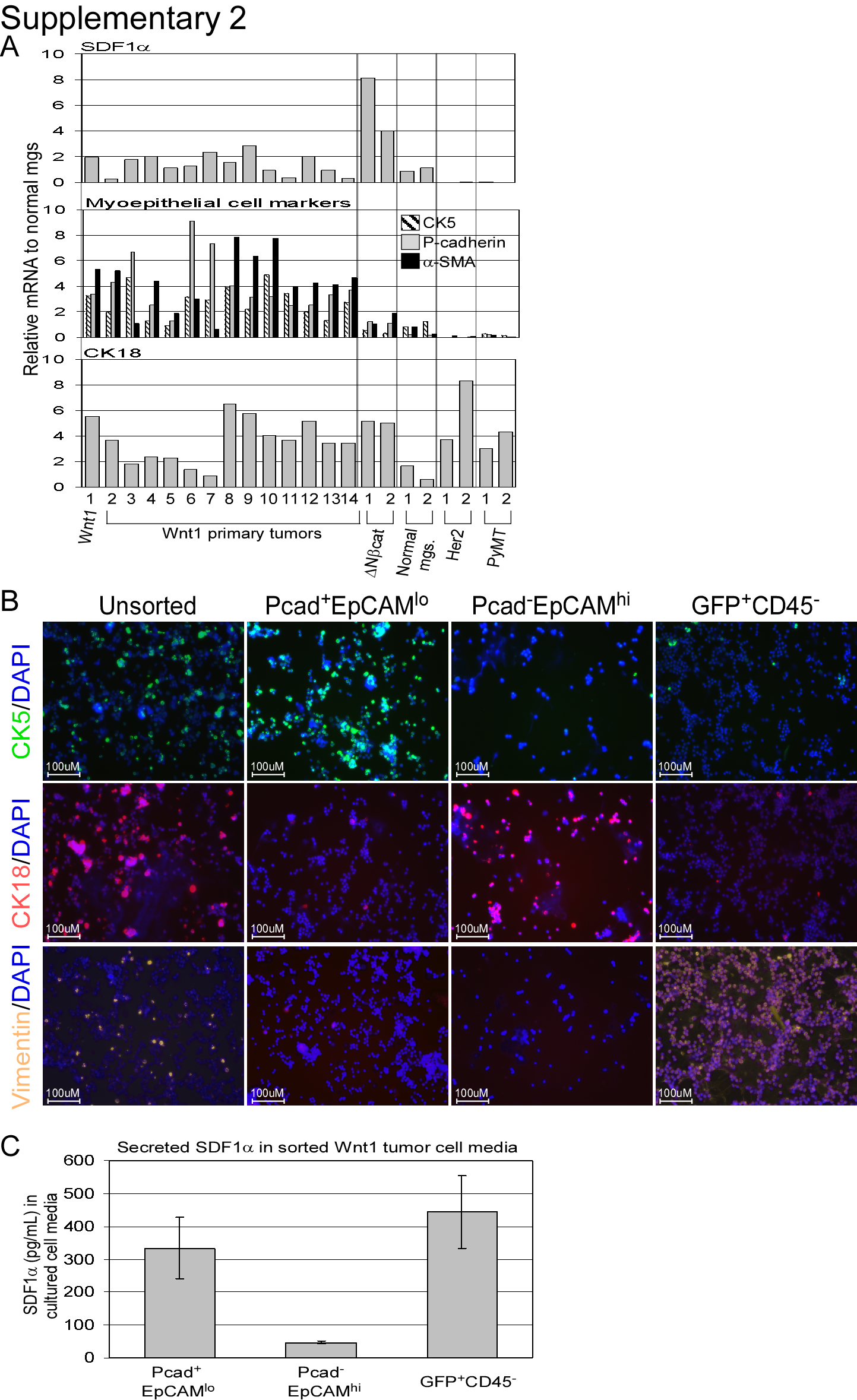

Supplement: Figure S2 — Determination of cells in Wnt1 tumors that produce SDF1. A, qRT-PCR analysis of SDF1α, myoepithelial cell, and luminal cell markers mRNA transcripts in various MMTV tumors. Myoepithelial cell maker transcripts are shown as a combined group: CK5 in stripped bars, P-cadherin in grey bars, and α-SMA in black bars. Primary Wnt1 tumors are as indicated and all of the other tumors are from passaged tumors. Relative mRNA level for each gene was normalized to average transcript levels of normal mammary glands. B, immunocytochemical analysis of the sorted Wnt1 tumor cell populations stained with anti-CK5 (green), -CK18 (red), and -vimentin (orange) antibodies, and DAPI stained nuclei (blue). Immunofluorescent staining was done on sorted cells by spotting sorted cells on glass slides, allowing cells to be dried onto the slides, then fixing cells with 4% paraformaldehyde for 10 minutes at room temperature, and permeabilizing cells with cold methanol for 10 minutes at 4oC. Fixed cells was blocked with MOM (Vector Laboratories), incubated with primary antibodies, followed by incubation with secondary antibodies. Finally, cells were washed and mounted by Prolong Gold (Invitrogen) containing DAPI dye. C, ELISA analysis of secreted SDF1α protein in media taken from cultured myoepithelial cells (Pcad+EpCAMlo), luminal cells (Pcad−EpCAMhi), and stromal fibroblasts (GFP+CD45-) sorted from Wnt1 tumors. Error bars represent standard deviations derived from triplicate plated cells. (10.21 MB TIF) [file pone.0008611.s002.tif]

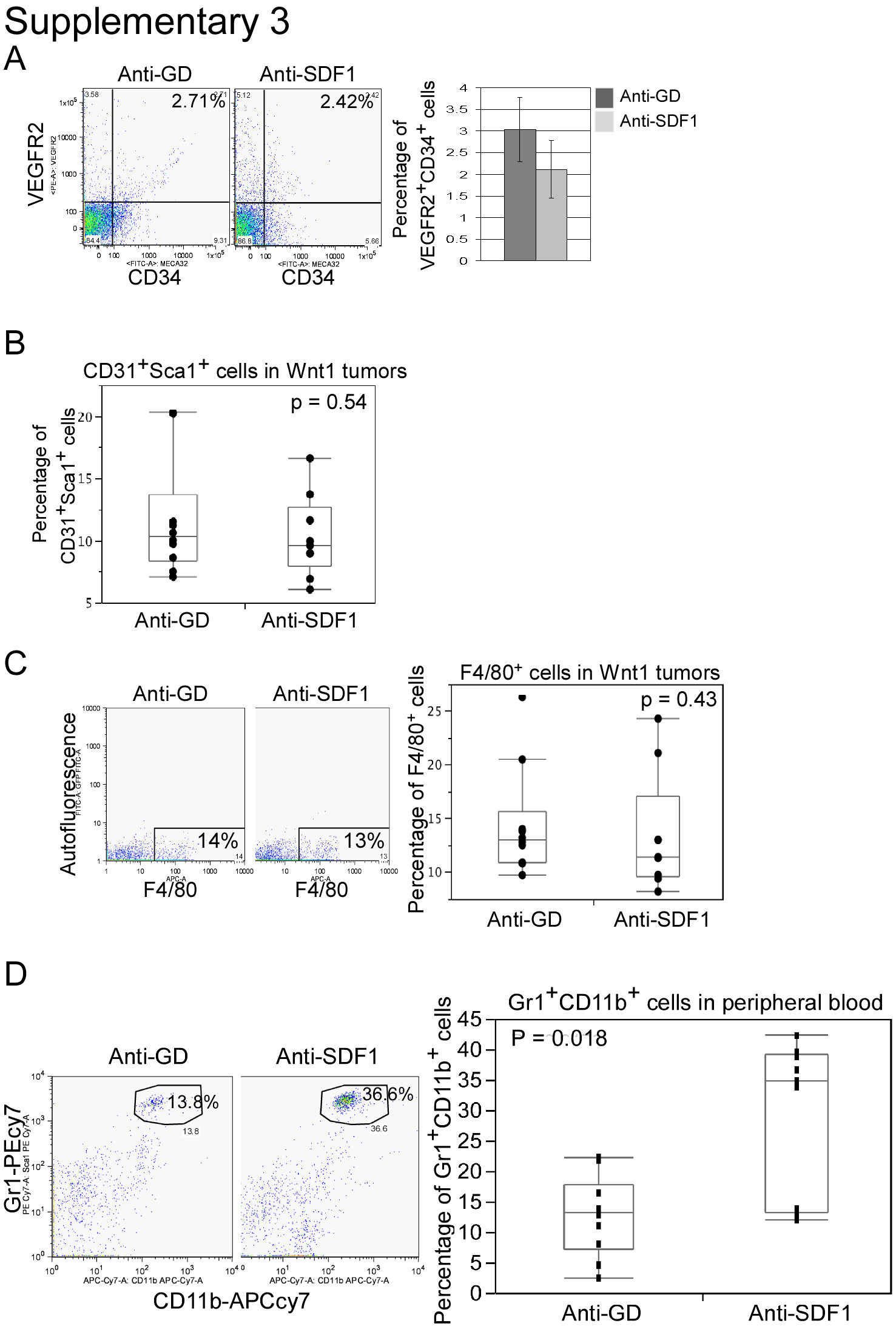

Supplement: Figure S3 — Assessment of various BMDCs that may contribute to tumor angiogenesis following anti-SDF1 treatment of Wnt1 tumors. FACS profiles and histogram/box plots of endothelial progenitor cells CD34+VEGFR2+ cells (A), CD31+Sca1+ cells (B), and tumor-associated macrophages (F4/80+; C) in the Wnt1 tumors following anti-SDF1 treatment of tumor-bearing mice. Indicated number in the FACS profile represents percentage of cells within the designated gate. P value was calculated by two-sided Wilcoxon Rank-Sum test, and error bars represent standard deviations from three independent FACs analysis. D, FACS profiles and box plots of Gr1+CD11b+ cells in the peripheral blood of Wnt1 tumor-bearing mice (n = 10) following anti-SDF1 or control anti-GD treatment. (9.68 MB TIF) [file pone.0008611.s003.tif]

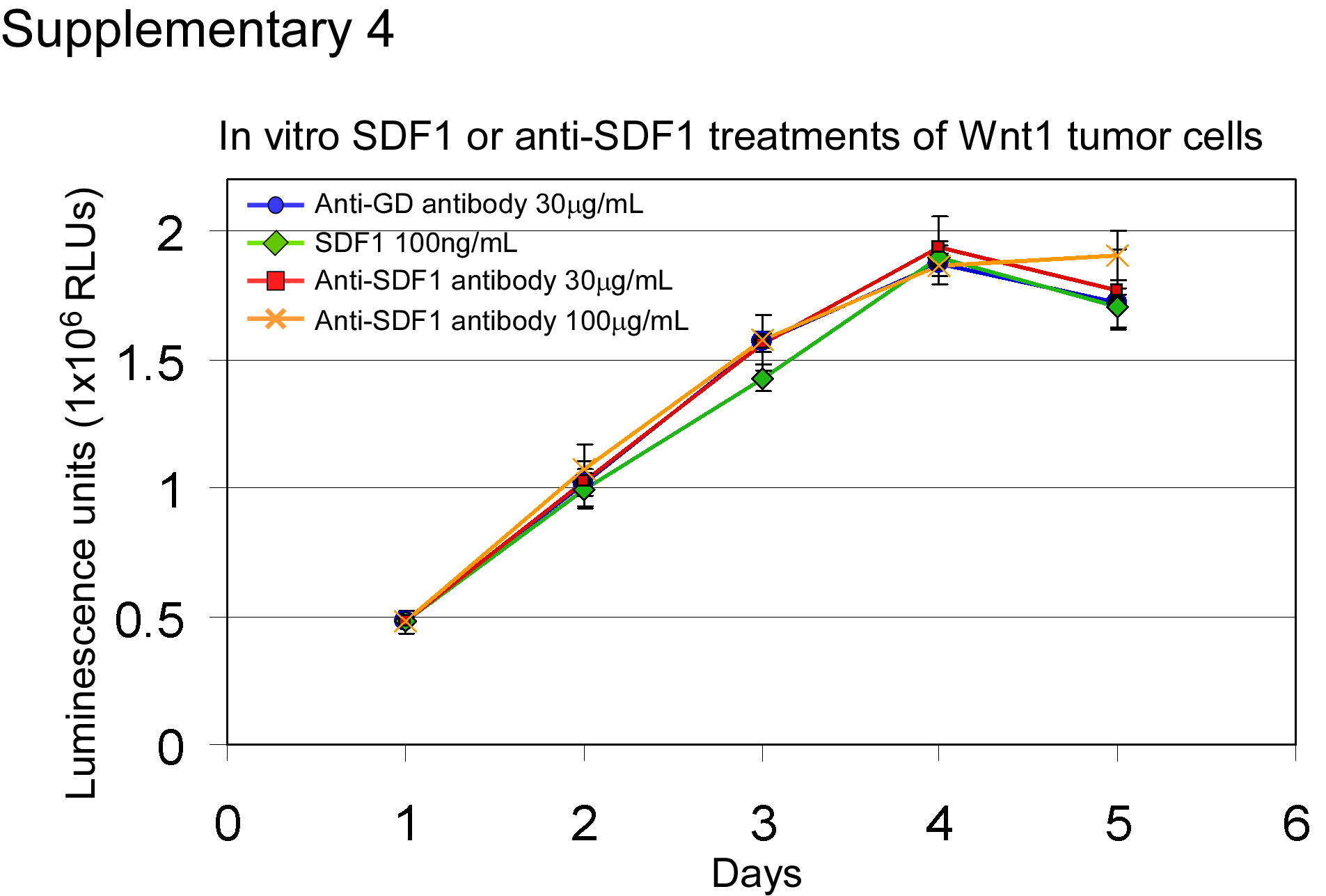

Supplement: Figure S4 — Wnt1 tumor cells treated with purified SDF1 and anti-SDF1 antibody. Dissociated Wnt1 tumor cells were cultured in media containing control anti-GD antibody (30 µg/mL; blue), SDF1 (100ng/mL; green) or various amounts of anti-SDF1 antibody (30 µg/mL in red or 100 µg/mL in orange). Cell viability was measured everyday following treatments, and average cell viability (RLUs) was calculated from three independently plated cells. Error bars represent standard deviations. (7.36 MB TIF) [file pone.0008611.s004.tif]
